# The Modulation Effect of MoS_2_ Monolayers on the Nucleation and Growth of Pd Clusters: First-Principles Study

**DOI:** 10.3390/nano9030395

**Published:** 2019-03-08

**Authors:** Ping Wu, Min Huang, Naiqiang Yin, Peng Li

**Affiliations:** 1 School of Electrical and Electronic Information, Shangqiu Normal University, Shangqiu 476000, China; wup@mail.ustc.edu.cn (P.W.); yinnq@mail.ustc.edu.cn (N.Y.); 2 School of Physics and Engineering, Zhengzhou University, Zhengzhou 450001, China; 3 Faculty of Physics and Electronic Sciences, Hubei University, Wuhan 430062, China

**Keywords:** Pd clusters, initial growth, work function, diffusion, first principles calculations

## Abstract

The geometries, electronic structures, adsorption, diffusion, and nucleation behaviors of Pd*_n_* (*n* = 1–5) clusters on MoS_2_ monolayers (MLs) were investigated using first principles calculations to elucidate the initial growth of metal on MoS_2_. The results demonstrate that Pd clusters can chemically adsorb on MoS_2_ MLs forming strong Pd–S covalent bonds with significant ionic character. We investigated the initial growth mode of Pd clusters on MoS_2_ monolayers and found that Pd*_n_* clusters tend to adopt pyramid-like structures for *n* = 4–5 and planar structures lying on MoS_2_ substrates for *n* = 1–3. It can be explained by the competition between adsorbate–substrate and the intra-clusters’ interactions with the increasing coverage. Compared with pristine MoS_2_ MLs, the work function was reduced from 5.01 eV upon adsorption of Pd monomer to 4.38 eV for the case of the Pd_5_ clusters due to the charge transfer from Pd clusters to MoS_2_ MLs. In addition, our calculations of the nucleation and diffusion behaviors of Pd clusters on MoS_2_ MLs predicted that Pd is likely to agglomerate to metal nanotemplates on MoS_2_ MLs during the epitaxial stacking process. These findings may provide useful guidance to extend the potential technological applications of MoS_2_, including catalysts and production of metal thin films, and the fabrication of nanoelectronic devices.

## 1. Introduction

In recent years, two-dimensional (2D) layered transition metal dichalcogenides (TMDs), particularly MoS_2_, exhibiting excellent electronic and optical properties, have drawn great attention due to their potential applications in flexible nanoelectronics, photonic devices, memory devices, etc. [1,2,3,4]. A series of experimental and theoretical studies have confirmed that MoS_2_ monolayers (MLs) decorated with metal nanoparticles (NPs) could potentially extend its functionalities as novel catalysts, spintronic devices, and thermoelectric and photoelectric materials, which is owing to the unique size-dependent properties of metal nanoparticles [5,6,7,8]. For instance, Chen et al. [5] reported the metal clusters (Pd, Pt, and Ag) supported on MoS_2_ MLs tend to display excellent electrocatalytic activity compared to those on graphene. Fu et al. [6] found that Au nanoparticles on two-dimensional MoS_2_ nanosheets can be used to fabricate an attractive alternative photoanode for efficient photoelectron chemical miRNA detection. Recently, Burman and co-workers [7] successfully fabricated Pt decorated MoS_2_ nanoflakes, and further confirmed its potential application as the sensing layer of an ultrasensitive resistive humidity sensor. Besides, Li et al. [9] reported that Au NPs imposed remarkable p-doping effects onMoS_2_ transistors, which implied that a controllable method of metal NP decoration provides an effective way to design future optoelectronic devices. Furthermore, Guo et al. [10] used 2D MoS_2_ effectively decorated with Au nanoparticles to improve the performance of flexible thermoelectric materials, which may become an alternative material for wearable thermoelectric devices. In addition, the MoS_2_–Pd nanoparticle hybrid structure was used to engineer the oxide/electrode interface of hafnium oxide (HfO*_x_*)-based metal oxide-based, resistive random-access memory, which has huge potential application in the field of data storage and wearable electronics [11]. 

Meanwhile, both experimental and theoretical studies have revealed that the surfaces of MoS_2_ MLs with graphene-like structures can play an active role as a host surface for the clusterization and nucleation characteristics of transition metal atoms. Huang and co-workers [12] demonstrated that MoS_2_ nanosheets can be used to direct the epitaxial growth of Pd, Pt, and Ag nanostructures by wet-chemical synthetic method under ambient conditions in experiments. Song et al. [13] studied the nucleation and growth dynamics of Au nanoparticles on MoS_2_ nanoflakes by in situ liquid-cell transmission electron microscopy (TEM). In order to design more efficient and less expensive catalysts, the evolution of morphology and epitaxial growth of Pt NPs on MoS_2_ (001) surfaces was systematically analyzed by density functional theory study [14]. Recently, Jiang and co-workers [15] proposed that 2D Fe/MoS_2_ heterostructures constructed by deposition of Fe atoms on MoS_2_ exhibits robust half-metallic magnetism and possesses robust ferromagnetic and half-metallic properties with 100% spin-filter efficiency based on first-principles calculations. Similarly, Cooley et al. [16] showed that graphene/MoS_2_ heterostructures can be used as templates to grow stable clusters lying planar to the surface, as well as to prepare monoatomic layers of ordinary metals.

It is also known that the nature of metal–semiconductor interfaces plays a more important role than MoS_2_ MLs themselves in MoS_2_-based optoelectronics and nanoelectronics [17]. Motivated by the high work function of Pd and the small lattice mismatch of Pd and MoS_2_, Pd can be used as the p-type metal contact on MoS_2_ to modify the Schottky barrier height (SBH) and the charge carrier injection rates. Fontana et al. [18] have observed that MoS_2_-based transistors show hole-doping and electron-doping behaviors when Pd and Au are used for source and drain contacts, respectively, and the formation of Schottky junctions at contact interfaces remarkably induced a clear photovoltaic effect. Later work highlighted an epitaxial growth mode of Pd deposition on MoS_2_ bulk surface and a strong band bending effect and high contact resistance were observed for the Pd/MoS_2_ interface [19]. Although metal contact engineering is a very useful avenue for building high-performance MoS_2_-based devices, there is little research on how MoS_2_ MLs modulate the nucleation and growth processes of Pd NPs and which further explores the nature of Pd*_n_*/MoS_2_ interfaces. Above all, it is crucial to explore the formation and diffusion properties of Pd clusters on MoS_2_ MLs and to investigate the modulation effect of MoS_2_ surfaces on the growth of Pd*_n_* clusters for the sake of improving deposition technological applications at the device level.

In this work, we aim to systematically investigate the adsorption behaviors including geometries, relative stability, and electronic properties of Pd*_n_* (*n* = 1–5) clusters on MoS_2_ MLs, and the diffusion behaviors of Pd*_n_* (*n* = 1–5) clusters on MoS_2_ are discussed using density functional theory (DFT) calculations. The rest of the paper is organized as follows. In Section 2 we briefly describe the calculation method used. In Section 3 we report our results and discussions including the structures, relative stabilities, and the electronic properties of Pd clusters supported by MoS_2_ MLs. Meanwhile, in order to better understand the role of MoS_2_ surfaces during the nucleation of Pd clusters, the diffusion characteristics of Pd*_n_* (*n* = 1–5) clusters are discussed in detail. The conclusions are given in the last section.

## 2. Computational Details

The geometries, electronic structures, and diffusion characters of Pd*_n_* (*n* = 1–5) clusters absorbed at MoS_2_ MLs were carried out with the first-principles calculations based on density functional theory (DFT) under the generalized gradient approximation (GGA) as implemented in the VASP code [20,21]. A plane wave basis set with the projector-augmented plane wave (PAW) was performed to describe the ion–electron interaction [22]. The plane-wave cutoff corresponding to a kinetic energy of 450 eV was adopted. The *k*-point meshes were generated according to the Monkhorst–Pack scheme [23] and 9× 9 × 1 mesh was used to sample the supercell which consisted of 4 × 4 MoS_2_ units in the calculations. In order to prevent the interactions between neighboring slabs, a thick vacuum layer of more than 15Å was adopted in a direction perpendicular to the surface. During structural relaxation, all the atomic coordinates (including Mo, S, and Pd) were fully relaxed until the Hellmann–Feynman forces were smaller than 0.01 eV/Å.

To describe quantitatively the energetic trends of adsorbed Pd clusters on the MoS_2_ ML and to further explore the modulation of MoS_2_ substrate on the growth mechanism of Pd clusters, we introduce adsorption energy *E_A_*, binding energy *E_B_*, and intra-cluster binding energy *E_IB_*, which are defined as the following:

(1) The adsorption energies *E_A_*,
  (1) $$E_{A}=\left[E_{{\mathrm{MoS}}_{2}}+E_{{\mathrm{Pd}}_{n}}-E_{tot}^{{\mathrm{Pd}}_{n}/{\mathrm{MoS}}_{2}}\right]/n,$$
  where $E_{tot}^{{\mathrm{Pd}}_{n}/{\mathrm{MoS}}_{2}}$ and $E_{{\mathrm{MoS}}_{2}}$ are the total energies of the MoS_2_ monolayer with and without Pd*_n_* clusters, $E_{{\mathrm{Pd}}_{n}}$ is that of the floating Pd*_n_* clusters consisting of *n* Palladium atoms. *E_A_* can be used to describe quantitatively the strength of the adsorbate–substrate interaction.
(2) The binding energy *E_B_*,
  (2) $$E_{B}=\left[E_{{\mathrm{MoS}}_{2}}+nE_{\mathrm{Pd}}-E_{tot}^{{\mathrm{Pd}}_{n}/{\mathrm{MoS}}_{2}}\right]/n,$$
  where $E_{\mathrm{Pd}}$ is the total energy of an isolated Palladium atom in a cubic supercell of 20 Å × 20Å × 20Å. The binding energy *E_B_* reflects the relative stability of the Pd*_n_* clusters supported by the MoS_2_ ML.
(3) The intra-cluster binding energy *E_IB_*,
  (3) $$E_{IB}=E_{B}-E_{A},$$
  which could qualitatively reflect the strength of the Pd–Pd interactions. In order to compare directly, all considered energy terms were normalized with respect to the number of Pd atoms, given per adatom.

To get insight to the nucleation mechanism and growth mode of Pd clusters, we also calculated the diffusion properties of Pd*_n_* (*n* = 1–5) clusters supported by the MoS_2_ ML. The diffusion barrier and transition states were determined from the minimum energy pathway by employing the climbing-image nudged elastic band (NEB) method [24,25]. All geometries were optimized until the maximum force in every degree of freedom was less than 0.005 eV Å.

## 3. Results and Discussions

### 3.1. Geometries and Stabilities for Pd_n_ (n = 1–5) Adsorbed at MoS_2_ ML

**Figure 1:**
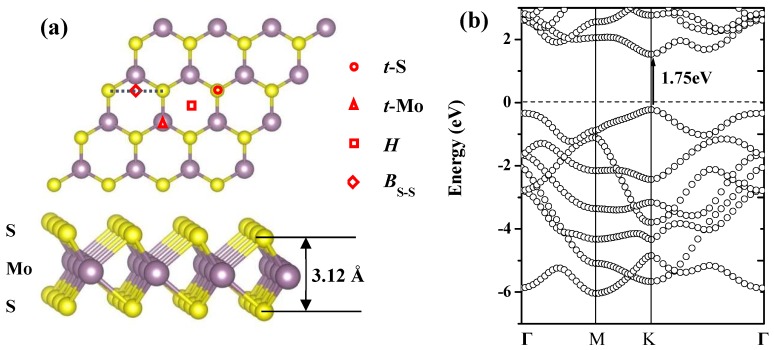
(**a**) Top and side views for the pristine MoS_2_ monolayer (ML); S and Mo atoms are in yellow (small) and lavender (large) spheres, respectively. (**b**) Band structure of pristine MoS_2_ monolayer and Fermi level is indicated by gray dashed line.

In order to understand the adsorption properties of Pd*_n_* (*n* =1–5) clusters and explore the nucleation mechanism and initial growth of Pd nanoparticles on MoS_2_ MLs, it is essential to identify the structural and electronic properties of pure MoS_2_ ML for comparison. Figure 1a shows the top and side view of a 4 × 4 supercell of the MoS_2_ ML; the calculated results show that the thickness of the MoS_2_ ML was 3.14 Å and the bond length of the S–Mo was 2.84 Å, which is consistent with previous studies [26,27]. Besides, we calculated the band structures of the MoS_2_ ML as shown in Figure 1b. The calculated results indicate that the MoS_2_ ML presented semiconducting character with a direct gap of 1.75 eV at the K point, which is in good agreement with the previous theoretical results of 1.70 eV [28] and experimental results of 1.80 eV [29].

We first investigated the geometries and adsorption properties of a single Pd adatom on the MoS_2_ ML. For the case of Pd monomer, we considered the binding of Pd on four high-symmetry sites: the hollow (*H*) site at the center of a hexagon, the top site directly above Mo (*t*-Mo) and S (*t-*S*)*, and the bridge (*B*_S–S_) site at midpoint of the S–S bond, as shown in Figure 1a. It is known that the larger the adsorption energy *E_A_*, the stronger the interaction between the adsorbate and substrate. Table 1 lists the structural parameters and adsorption energies for all considered adsorption configurations of Pd monomer adsorbed at the MoS_2_ ML. The calculated results show that the *t*-Mo site with the adsorption energy of 2.16 eV was the most energetically favorable location, which is consistent with previous studies [26,30]. The Pd monomer adsorbed at the *t-*S and *H* sites were 0.57 and 0.37 eV, respectively, less stable than that of the *t*-Mo adsorption configuration. Our calculated results show that Pd monomer located at the *B*_S-S_ site finally relaxed to the *t*-Mo configuration, which indicates that Pd monomer adsorbed at the *B*_S-S_ site was unstable. As summarized in Table 1, Pd bonds to the surrounding S atoms with a bond length of 2.34, 2.42, and 2.18 Å for the considered adsorption configurations of *t*-Mo, *t*-S, and *H*, respectively, which are comparable with those in two types of PdS_2_ monolayer with values of 2.34 and 2.40 Å [31]. The distances between Pd and nearest neighboring Mo are 2.34, 2.30, and 2.26 Å for three considered configurations, which are mainly resulting from the different adsorption sites. The Pd monomers located at 1.33, 1.59, and 2.19 Å higher than the underlying MoS_2_ surface for three considered adsorption configurations, which is consistent with the decreased trend for the calculated adsorption energies from the *t*-Mo to *t*-S configuration.

**Figure 2:**
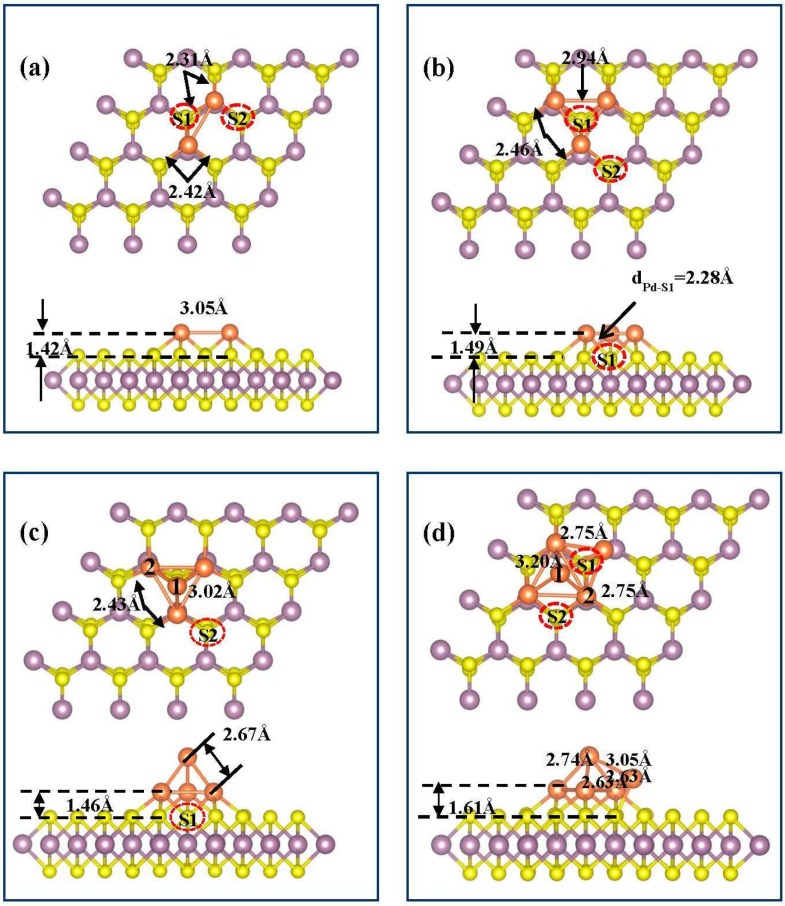
(**a**–**d**) Top and side views for most stable configurations of palladium dimer, trimer, tetramer, and pentamer adsorbed at the MoS_2_ monolayer, respectively. The yellow, lavender, and orange balls represent S, Mo, and Pd atoms, respectively.

**Figure 3:**
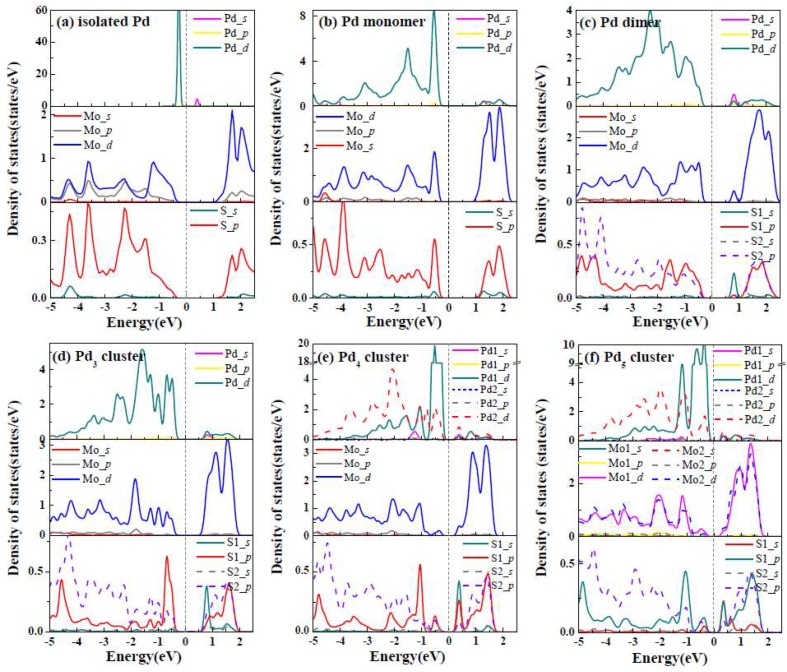
(**a**) Partial density of states (PDOS) of isolated Pd atoms and S and Mo of pristineMoS_2_ ML. (**b**–**f**) PDOS of Pd adatoms of Pd*_n_* (*n* = 2–5) clusters and surrounding S and Mo of underlying MoS_2_ substrate. The Fermi level was set to 0 eV and is represented by the dashed lines.

We have chosen several initial configurations to search the most stable configurations of Pd*_n_* (*n* = 2–5) clusters adsorbed at the MoS_2_ ML, which is shown in Figure 2. The calculated structural parameters and adsorption, binding, and intra-cluster binding energies for the lowest energy configurations of Pd*_n_* (*n* = 1–5) adsorbed on the MoS_2_ ML are summarized in Table 2. The most preferential configuration of Pd dimer adsorbed at the MoS_2_ ML was that two Pd atoms both adsorbed at the top of Mo, and they were separated by 3.05 Å, which was smaller than the calculated lattice constant of MoS_2_ ML, as shown in Figure 3a. For the cases of Pd_3_ cluster adsorbed at MoS_2_ ML, the calculated results show that the *t*-(Mo)_3_-S configuration with the largest adsorption energy of 1.44 eV presents higher stability than the *t*-(Mo)_3_-h configuration due to the extra binding of S1 atom, in which Pd trimer stands in a plane parallel to the MoS_2_ surface. Besides, it is not surprising that the considered *t*-(Mo)_3_-L with three Pd atoms in a line adsorbed at the top of Mo is higher in energy than the triangular islands because of the decrease in the number of intra Pd–Pd bonds. The average bond length of Pd–Pd and the height of Pd_3_ cluster above the MoS_2_ surface are 2.94 and 1.49 Å, respectively. The Δ–(Mo)_3_ structure where two Pd atoms were located at the *t*-Mo sites and the third one was located at the bridge site of the two Pd atoms was also considered for the case of the Pd_3_ cluster. However, different from the case of Pt_3_ on MoS_2_ ML, such vertical configuration is unstable as it is about 0.65 eV less stable than the *t*-(Mo)_3_-S configuration. For the case of Pd tetramers adsorbed at MoS_2_ surface, we considered three possible configurations, including aplanar-(Mo)_4_ (four Pd atoms located at the *t*-Mo site, not shown here), pyramid-like *t*-(Mo)_4_-h, and *t*-(Mo)_4_-S configurations. The computed results show that the most favorable structure is *t*-(Mo)_4_-h (shown in Figure 2c) for Pd tetramer adsorbed at the MoS_2_ ML, implying the growth mechanism transitions from a two-dimensional (2D) to a three-dimensional (3D) mode from the formation of Pd_4_ cluster. Based on the most stable configuration of Pd_4_ tetramers supported by the MoS_2_ ML, we considered three initial geometries for Pd pentamer. After full structural optimization, the most stable structure was asquare pyramid *t*-(Mo)_5_, shown in Figure 2d, which could be obtained by adding one additional Pd atom adsorbed at a neighboring *t*-Mo site with respect to the *t*-Pd_4_-h configuration.

From the above calculations, it is clear that the palladium clusters are energetically preferred to lying planar to the surface for the initial growth of Pd*_n_* clusters on MoS_2_ ML at very low coverage (from Pd_1_ to Pd_3_). With increasing the coverage, the Pd*_n_* clusters immediately form islands clusters, such that the morphology of *t*-(Mo)_4_-h and *t*-(Mo)_5_-h are the most stable configurations for Pd tetramer and pentamer, respectively. Therefore, under situations dominated by the thermodynamic effects, the clusters with planar structures may be expected to only appear in the very early growth stage and the size of these clusters are very small (e.g., Pd_2_ and Pd_3_), which is immediately followed by the Vomler–Weber growth mechanism.

In our previous study on the initial growth of Pd*_n_*/NiAl(110) [32], we reported that small-size Pd*_n_* (*n* = 1–5) clusters favor the planar structures on the NiAl(110) surface, which was explained by the stronger interaction between Pd*_n_* clusters and NiAl substrate than the interaction among Pd adatoms in clusters. In order to explore the modulation of MoS_2_ substrate on the growth mechanism of Pd*_n_* clusters, it is crucial to understand the evolutions of the metal–metal and metal–slab interactions with the increase of cluster size. We summarize the adsorption, binding, and intra-cluster binding energies as well as structural parameters for the most stable structures of Pd*_n_* (*n* = 1–5) adsorbed on the MoS_2_ ML in Table 2. It was found that the adsorption energies, *E_A_*, decreased from 2.16 to 0.86 eV when the Pd coverage increased from Pd_1_ to Pd_5_ clusters, which indicates the strength of the interactions between the Pd*_n_* (*n* = 1–5) clusters and MoS_2_ gradually weakened. The results are reasonable since the height of Pd clusters above MoS_2_ increases from Pd monomer to Pd_3_ cluster with planar structures. For the case of pyramid-like structures, Pd_5_ waslocated at 0.03 Å higher than that of Pd_4_ above the MoS_2_ ML as listed in Table 2; however, the additional Pd–Pd bonds weakened the Pd–S bonds resulting in less stability of Pd_5_ cluster. The binding energy, *E_B_*, that reflects the relative stability of the Pd*_n_* (*n* = 1–5)/MoS_2_ system, gradually increased from 2.21 eV for Pd_2_ to 2.38 eV for Pd_5_, which indicates the relative stability of larger Pd clusters adsorbed at the MoS_2_ ML was higher than that of smaller ones. With the increase of Pd coverage (from Pd_2_ to Pd_5_), the intra-cluster binding energy *E_IB_* rapidly increased from 0.37 to 1.52 eV, which suggests that the interaction among the Pd adatoms in the Pd_5_ cluster was stronger than those of smaller ones. Besides, it was noticeable that the *E_IB_* was larger than *E_A_* for Pd_4_ and Pd_5_, which indicated that intermetallic Pd–Pd bonds in clusters were stronger than the bonds between Pd and surrounding S or Mo. This can be used to explain the fact that the most stable structures for Pd_4_ and Pd_5_ started to appear around the three-dimensional structures with smaller Pd–Pd bond lengths (about 2.63 Å). Compared with a previous study on Pd*_n_* cluster/graphene [33], in which size-selected monodisperse nanoclusters were identified by scanning tunneling microscopy, Pd*_n_* clusters supported by MoS_2_ are more stable due to larger binding energies and shorter distance between Pd*_n_* and MoS_2_ substrate, which indicated that the MoS_2_ ML was inert and an ideal template for deposition of the metal NPs to some extent.

### 3.2. Electronic Properties of Pd_n_ (n=1–5)/MoS_2_ Monolayer

In order to better understand and control how the deposition of Pd clusters affect the structure and electronic properties of MoS_2_, we calculated the density of states (DOS) of MoS_2_ ML with and without the adsorption of Pd*_n_* (*n* = 1–5) clusters shown in Figure 3. It is clear that the 4*d* states of isolated Pd atom was very sharp at 0.35eV, while 4*d* states of Mo hybrids with the 3*p* states of neighboring S atoms for pure MoS_2_ ML. Upon the adsorption of Pd monomer on MoS_2_ (Figure 3b), the band gap decreased to about 1.09 eV, which is mainly attributed to the hybridization of the 4*d* states of Pd atoms with 4*d* states of underlying Mo and 3*p* states of the nearest surrounding S at 0.50 eV below Fermi level. For the case of Pd dimmer adsorbed at MoS_2_ ML (Figure 3c), a gap state emerged at the 0.80 eV above the Fermi level, which resulted from the hybridizations between Pd atoms and the nearest-neighboring Mo and S atoms. For the case of Pd_3_ cluster, the partial density of states (PDOS) of three Pd adtoms were similar due to the identical atomic environments as shown in Figure 3d. We found that Pd adatoms hybridize more strongly with the S atom located at the hollow site of the Pd trimer (labeled by S1 in Figure 2b) than the nearest-surrounding S atoms (labeled by S2 in Figure 2b) at −0.80 eV. For the case of the Pd_4_ and Pd_5_ clusters with pyramid-like geometries (Figure 3e–f), the DOS of the topmost Pd adatoms labeled as Pd_4_-1 and Pd_5_-1 (located at the second layer of clusters) in Figure 2c were relatively localized compared with those of the underlying Pd (labeled by Pd_4_-2 and Pd_5_-2 in Figure 2d, which were located at the first layer of the clusters), which indicated that the electronic properties of the topmost Pd were hardly affected by the MoS_2_ substrate. The calculated results also show that the gap state located above the *E_F_* was shifting close to 0 eV from the Pd_3_ to Pd_5_ clusters, which caused the band gap to decrease from 0.70 eV of the Pd_3_/MoS_2_ system to 0.19 eV of the Pd_5_/MoS_2_ system.

### 3.3. Charge Redistribution and Work Functions

**Figure 4:**
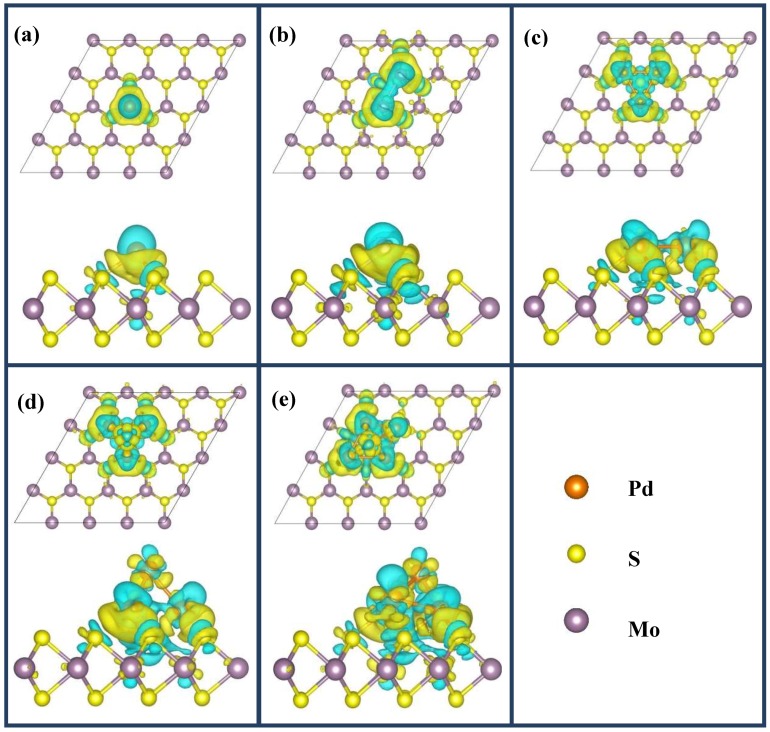
(**a**–**e**) Charge density difference plots for the most stable configurations Pd*_n_* (*n* = 1–5) clusters adsorbed on the MoS_2_ monolayer. Top and bottom images show the top and side views of adsorption configurations, respectively. Yellow regions represent charge accumulation, and blue regions show charge loss. The iso-surface value was0.001 e Å^−3^.

In order to analyze the character of bonds between adsorbates and MoS_2_ substrate as well as the charge redistribution of MoS_2_ upon the adsorption of Pd clusters, we calculated and analyzed the electron density difference, which is defined as $\Delta \rho =\rho_{{\mathrm{Pd}}_{n}-{\mathrm{MoS}}_{2}}-\rho_{{\mathrm{MoS}}_{2}}-\rho_{{\mathrm{Pd}}_{n}}$, where $\rho_{{\mathrm{Pd}}_{n}-{\mathrm{MoS}}_{2}}$ is the charge density of total system, $\rho_{{\mathrm{MoS}}_{2}}$ and $\rho_{{\mathrm{Pd}}_{n}}$ are the charge densities of pristine MoS_2_ ML and the free-floating Pd clusters in the frozen geometry they adopted on the Pd*_n_*/MoS_2_ system, respectively. Figure 4 shows the corresponding difference in electron densities for all considered optimized stable configurations, yellow and blue region represent charge accumulation and charge loss, respectively. As shown in Figure 4, the charge redistributions upon the deposition of Pd*_n_* cluster mainly involved Pd clusters and surrounding S and Mo atoms, which imply strong charge transfers between the Pd*_n_* (*n* = 1–5) clusters and MoS_2_ substrate. It is clear that there was strong electron density accumulation between Pd atoms and nearest-neighboring S atoms, which indicates that the bonds between Pd adatoms and surface S atoms present a covalent bond with partial ionic features. There are depletion regions close to the Pd atoms along the bond directions of Pd–S, which can be explained by the stronger electronegativity of S than Pd. For the cases of Pd_2_ and Pd_3_ clusters, the Pd atoms highly hybrid with the center-S (labeled as S1 in Figure 2). It is also observed that the characteristics of Pd–Pd bond remain strong metallic upon Pd clusters supported by MoS_2_ ML. However, for the cases of the Pd_4_ and Pd_5_ clusters (Figure 4d,e), it is surprising that strong electron density accumulation was found for the topmost Pd atoms (such as Pd_4_-1 and Pd_5_-1), which indicates that the top Pd atoms directly receive charge from the underling Pd layers rather than losing charge to the MoS_2_ substrate.

To further give a detailed insight into the charge transfer, we also calculated the atomic populations for the most favorable configurations of Pd clusters adsorbed at the MoS_2_ ML, as summarized in Table 3. Upon Pd adatoms adsorbed at the *t-*Mo site, Pd adatoms lost about 0.26e to the MoS_2_ ML by Bader analysis, which suggested Pd–S bonds exhibited a relatively significant ionic bonding component, as illustrated by the substantial charge density difference between Pd and neighboring S atoms shown in Figure 3a. Similarly, Pd adatoms in dimer and trimer averagely contributed about 0.20 and 0.18e to the MoS_2_ ML, respectively. However, in the cases of Pd_4_ and Pd_5_ clusters with pyramid-like geometry, Pd atoms in the first layer and second layer of the clusters behave in a different way. The Pd atoms in the second layer of the cluster (Pd_4_-1 and Pd_5_-1) obtained charge, while the Pd atoms of the first layer lost charge. This result is in good agreement with the phenomenon of charge accumulation near the topmost Pd atoms as shown in Figure 4d,e. Therefore, it is not surprising that the MoS_2_ slabs obtained less charge from Pd_4_ and Pd_5_ clusters than that of the Pd_3_ cluster.

**Figure 5:**
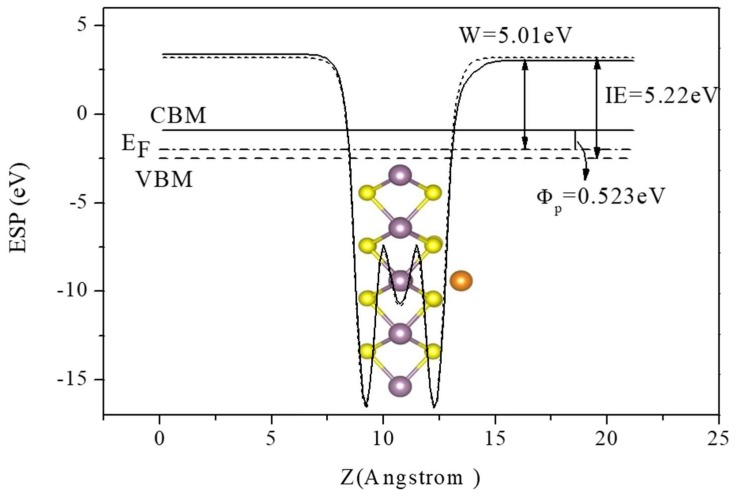
In-plane averaged electrostatic potential (ESP) for the MoS_2_ surface adsorbed with isolated Pd atom (solid line) and pristine MoS_2_ (dotted line). The values of work function (*W*), ionization energy (*IE*) for the MoS_2_ surface, and *p*-SBH (*Φ_P_*) are indicated.

Figure 5 shows the in-plane averaged electrostatic potential (ESP) for the MoS_2_ surface with Pd monomer (solid line) and that of the pristine MoS_2_ surface (dotted line), respectively. The same were done for other Pd clusters, which are not shown here. Ionization energy (IE), defined as the energy difference between the vacuum level and valence band maximum (VBM), was determined to be 5.48 eV for the clean MoS_2_ ML. Upon the Pd*_n_* (*n* = 1–5) clusters adsorption, the increase of ionization energy (Δ*I*) was observed, which can be quantificationally given by the energy difference between the vacuum levels. In addition, the work function is described as the following equation: *W* = *E_vac_*−*E_F_,* where *E_vac_* is the electrostatic potential in the vacuum region of the adsorbate side of the MoS_2_ surface, while *E_F_* refers to Fermi energy. The work function of pristine the MoS_2_ ML was estimated to be 5.26 eV, which is slightly higher than the experimental result of 5.03 eV [34].

As indicated in Figure 5, we also defined the energy difference between Fermi level (*E_F_*) and the CBM of the MoS_2_ ML as *p*-SBH (*Φ_P_*) for convenience, although the well-defined Schottky barrier contact had not formed yet in our considered initial growth stage of Pd clusters. In Table 4 we summarized the calculated work function (W), *p*-SBH (*Φ_P_*), dipole moments (*D_i_*), and the variation of ionization energy (Δ*I*) for the adsorption of Pd clusters. It became clear that the increasing of Pd coverage leads to decreases in the work function from 5.01 eV of Pd adatom to 4.38 eV of Pd_5_ cluster, which may result from the larger amount of CT from Pd*_n_* cluster to MoS_2_ as listed in Table 3. We think the variations of *p*-SBH are mainly attributed to the gap states caused by Pd adatoms (as shown in PDOS in Figure 3) and the partial charge transfer from 1stlayers to the second layer Pd atoms. The trend for the variations of ionization energy is obvious: as the cluster size increases (from Pd_1_ to Pd_5_), Δ*I* reduces rapidly, while Δ*I* rises in the initial growth stage of Ni clusters supported by MgO (001) [35]. In addition, the dipole moments (*D_i_*) were calculated by the product of transfer charge and the distance of adsorbate-substrate. The calculated dipole moments of Pd*_n_*/MoS_2_ were not in a monotonic variation, which is different from the monotonic increase of *D_i_* with the increase of Ni coverage on MgO (001).We think that the main reason for these distinct phenomena of the two systems is that the Pd*_n_* clusters injected electrons into the VBM of the MoS_2_ substrate while the Ni*_n_* clusters extracted electrons from the MgO (001) surface. Besides, Pd adatom, Pd_2_, and Pd_3_ clusters prefer the planar structures as discussed above, and there is only the interface dipole contribution in such systems. For the cases of Pd_4_ and Pd_5_ clusters with pyramid-like structures, intra-cluster dipoles due to the CT between the topmost layer and the lower part of the cluster also contribute to the dipole moments. Since these two dipoles have opposite directions, the dipole interactions between Pd_4_ or Pd_5_ clusters and MoS_2_ substrate can be cancelled to some extent.

Previous studies have confirmed that the coverage-dependent depolarization effects play a non-negligible role in metal–organic interfaces [36,37,38]. In order to get insight into the coverage-dependent depolarization effects caused by the interaction between the adjacent supercells, we also used a (6 × 6) supercell to study the adsorption of Pd*_n_* clusters at the MoS_2_ substrate. We found that the CT and work function of Pd*_n_* (*n* = 1–3)/MoS_2_ systems have no significant change, while the variation on CT and work function for Pd_4_/MoS_2_ and Pd_5_/MoS_2_ were less than 0.04 and 0.01 eV, respectively. Therefore, the interactions between Pd*_n_* (*n* = 1–5) clusters within adjacent supercells is ignorable, and thus the coverage-dependent depolarization effects can be ignored in Pd*_n_*/MoS_2_ considered in this work. The discrepancy between metal–organic interfaces [36,37,38] and Pd*_n_*/MoS_2_ interface maybe explained by different interactions between adsorbates and substrate and different sizes of adsorbates, such as non-specific bonding between organic molecules and substrates versus the covalent Pd–S chemical bonds in our study, and larger diameters of organic molecules than that of metal clusters.

### 3.4. Diffusion of Pd Clusters on an MoS_2_ Monolayer

**Figure 6:**
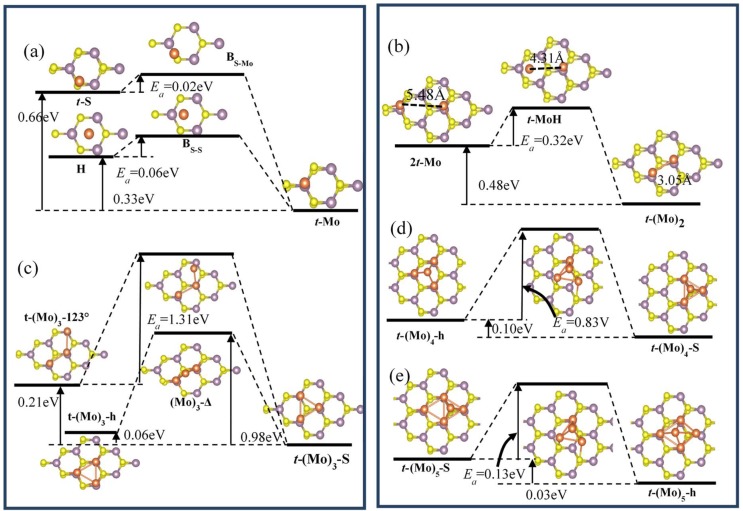
(**a**–**e**) Energy diagrams for the transformation between considered configurations of Pd*_n_* (*n* = 1–5) clusters on MoS_2_ surfaces. (**a**) The diffusion of a Pd adatoms from *t*-S and *H* sites to the most favorable *t*-Mo site, and (**b**) the nucleation process of Pd dimer on MoS_2_ surface. (**c**–**e**) The transformation to the most stable energy configurations of Pd*_n_* (*n* = 3–5) clusters on MoS_2_ surfaces.

In order to investigate the nucleation mechanism and diffusion properties of Pd clusters on the MoS_2_ ML, we analyzed the diffusion and surface mobility of Pd*_n_* (*n* = 1–5) clusters on the MoS_2_ ML from the most favorable adsorption configurations. We defined the reaction rate *k* as the following:

(4) $$k=v_{0}\exp\left(-\frac{\Delta E_{a}}{k_{B}T}\right),\;$$

where $v_{0}=10^{13}s^{-1}$ is the rate pre-factor, assumed to be irrelevant to the reaction or the hopping events, and *k_B_* and *T* are the Boltzmann constant and temperature, respectively. The equation indicated that reaction rate *k* is proportional to temperature *T*, while *k* is inversely related to the activation energy barrier $\Delta E_{a}$. In here, the activation energy barrier $\Delta E_{a}$ is computed from the total energy difference between the initial configuration and the saddle points of the minimum energy pathway between two adsorption configurations. The *k* value is usually used to measure the possibility of nucleation and diffusion by overcoming the energy barrier at the low Pd coverage. The energy diagrams of Pd*_n_* (*n* = 1–5) clusters nucleated or diffused on the MoS_2_ ML are shown in Figure 6. As shown in Figure 6a, the calculated activation energies were 0.12 and 0.02 eV for the on-surface diffusion of Pd monomer along the two paths of *t*-S→*t*-Mo and H→*t*-Mo, respectively, which indicates the diffusion of Pd monomer on the MoS_2_ surface show remarkably anisotropy. The activation energy barrier was much smaller than the value of 0.34eV for Pd monomer diffusion on MgO (100) via single atom hops between oxygen sites [39], implying the Pd adatom on the MoS_2_ surface was mobile during the in situ growth by deposition. According to Equation (4), the number of hopping events per second between two *T*_Mo_ sites was estimated to be 10^3^ s^−1^ along the Mo–H–Mo path and 10 s^−1^ along the Mo–S–Mo path at room temperature, respectively. The results indicated that the Pd adatoms are more likely to be mobile for coarsening and growth of large clusters rather than a random dispersion during the deposition. We also found that a newly deposited Pd atom prefers to bond to existing Pd adatoms located at *t-*Mo sites by possible diffusion, which is illustrated in Figure 6b. The energy barrier for the attachment of a Pd adatom to an existing Pd monomer to form Pd dimer is 0.32 eV, and the process is downhill by 0.48 eV. In other words, the deposited Pd adatoms are expected to bond to Pd monomer adsorbed at MoS_2_ rather than adsorb at a remote site. According to Equation (4), the higher the reaction temperature is, the easier the reaction occurs. For instance, the reaction rates *k* for the nucleation of Pd dimer from two Pd adatoms on MoS_2_ surface will increase about 140 and 485 times compared to that of room temperature (RT)under *T* = 500 and 600 K, respectively.

The three flat trimers *t*-(Mo)3-123°, *t*-(Mo)_3_-h, and *t*-(Mo)_3_-S shown in Figure 6b are easily accessible during metal deposition, which can be formed by the low-energy diffusion of Pd monomer on MoS_2_ surfaces to reach a Pd dimer. As shown in Figure 6b, although the total energies of the initial states of *t*-(Mo)3-123° and *t*-(Mo)_3_-h configurations were about 0.21 and 0.06 eV higher than that of the most stable configuration of *t*-(Mo)_3_-S due to the extra binding of the S1 atom, the energy barriers for transforming the flat *t*-(Mo)3-123° and *t*-(Mo)_3_-h configurations to *t*-(Mo)_3_-S were 1.31 and 0.92 eV, while the reverse processes were examined to surmount the energy barriers of 1.52 and 0.98 eV, respectively. We found that the transition state from *t*-(Mo)_3_-h to *t*-(Mo)_3_-S was Δ-(Mo)_3_, which was0.96 and 0.98 eV energetically higher than that of the initial and final states. However, the identical Δ-(Mo)_3_ configuration was predicted as the intermediate state with the lowest energy during the transformation between *t*-(Mo)_3_-h and *t*-(Mo)_3_-S configurations of Pt_3_ clusters on MoS_2_ ML as reported in Reference [14]. Such discrepancy may be due to the larger cohesive energy of Pt than that of Pd, which makes it is more likely to form islands for Pt NPs than Pd NPs.

We have considered various possible structures of palladium tetramer_,_ which is formed by the simple extension from *t*-(Mo)_3_-S and *t*-(Mo)_3_-h via the attachment of Pd monomer to Pd_3_ clusters. The computed results show three-dimensional *t*-(Mo) _4_-S was favored over *t*-(Mo) _4_-h only by 0.10 eV in total energy, both of which can be nucleated on the MoS_2_ ML. It was clearly observed that transition from *t*-(Mo) _4_-h to *t*-(Mo) _4_-S should overcome the energy barrier of 0.83 eV, which is twice that of the transformation barrier of Pt_4_ clusters with the identical structures on the MoS_2_ ML [14]. It indicated that the diffusion of Pt_4_ clusters was likely to be much more accessible compared with Pd_4_ clusters on MoS_2_ MLs. Similarly, the most preferable configuration can be accessed from configurations *t*-(Mo_4_)-h and *t*-(Mo_4_)-S by deposition of new Pd atoms or diffusion of Pd monomers to the nearest-neighboring *T*_Mo_ site. The two pyramid-like Pd_5_ clusters labeled as *t*-(Mo)_5_-h and *t*-(Mo)_5_-S are shown in Figure 6e, respectively, which were found to be stable and have a slight energy difference of about 0.03 eV. The computed results show that the transformation of *t*-(Mo)_5_-h and *t*-(Mo)_5_-S was almost barrier less with *E_a_* about 0.13 eV.

The diffusion of Pd atoms from the topmost site of a pyramid-like structure to the MoS_2_ substrate was a key factor to decide the growth mode of the Pd*_n_* cluster on the MoS_2_ monolayer. Therefore, we also calculated the diffusion behaviors of Pd atoms from topmost sites of Pd_4_ and Pd_5_ clusters to the MoS_2_ substrate. The calculated results show the topmost Pd atoms moving to the nearest-neighboring *t*-Mo sites of the MoS_2_ substrate needed to overcome the energy barriers of 0.73 and 1.13 eV for Pd_4_/MoS_2_ and Pd_5_/MoS_2_ systems, respectively. The relative high energy barriers imply that the Pd atoms prefer to form a sheet-supported metal nanotemplate on MoS_2_ in the initial growth stage, which is in conformity with the previous experimental study reported by Gong et al. [40] that Pd forms a uniform contact by physical vapor deposited on MoS_2_ monolayers.

## 4. Conclusions

In summary, we investigated theoretically the stable configurations, electronic structures, and surface mobility of Pd*_n_* (*n* = 1–5) clusters on MoS_2_ monolayers using first principles density functional theory calculations. The results demonstrate that Pd clusters can chemically adsorb on MoS_2_ MLs and Pd adatoms are strongly bound to the surface S atoms, which exhibit covalent bonds with significant ionic character. The geometries of Pd*_n_* cluster varies from a planar structure to a pyramidal morphology when the cluster size increases due to the relative strengths between Pd*_n_*–MoS_2_ and Pd–Pd interactions. Upon the deposition of Pd clusters, the band gaps of MoS_2_ weretunable due to the hybridization between 4*d* electrons of Pd and 3*s* electrons of S. The work function was modulated from 5.01 to 4.38 eV with the increase of Pd coverage, which resulted from the charge transfer from Pd clusters to MoS_2_ ML.

In addition, we investigated the nucleation and diffusion properties of Pd*_n_* (*n* = 1–5) clusters on MoS_2_ ML, e.g., the distant isolated Pd atoms or additional adatoms favor migrating to nearby Pd*_n_* clusters, which indicated that Pd is likely to agglomerate to metal nanotemplates on the MoS_2_ ML during the epitaxial stacking process. These findings may provide useful guidance to extend the potential technological applications of MoS_2_, including catalysts and production of metal thin films, and the fabrication of nanoelectronic devices.

## Figures and Tables

**Table 1:** The structural parameters (Å) and calculated adsorption energies *E_A_* (eV) for Pd monomer adsorbed at four high-symmetry sites of MoS_2_ monolayer.

Site	*d*_Pd–Mo_	*d*_Pd–S_	*d*_Mo–S_	*h*_Pd-sub_	*E_A_*
*t*-Mo	2.83	2.34	2.40	1.33	2.16
H	3.64	2.42	2.43	1.59	1.79
*t*-S	4.18	2.18	2.43	2.19	1.59
*B*_S-S_	–	–	–	–	–

**Table 2:** Calculated distance between Pd clusters and MoS_2_ surface (*d*_Pd-substrate_), bond length of Pd–S (*d*_Pd–S_), Pd–Pd distance (*d*_Pd–Pd_), adsorption energy *E_A_*, binding energy *E_B_*, and intra-cluster binding energy *E_IB_* for the lowest energy Pd*_n_* (*n* =1–5) configurations on the MoS_2_ ML. The units of structural parameters and energies are Å and eV, respectively.

Configurations	Pd	Pd_2_	Pd_3_	Pd_4_	Pd_5_
	*t*-Mo	*t*-(Mo)_2_	*t*-(Mo)_3_-S	*t*-(Mo)_4_-S	*t*-(Mo)_5_-h
***d*_Pd-substrate_**	1.33	1.42	1.49	1.46	1.44
***d*_Pd-S_**	2.34	2.31, 2.35, 2.42	2.28, 2.46	2.43, 3.49	2.33, 2.42, 2.47, 2.51
***d*_Pd-Pd_**	–	3.05	2.94	2.67, 3.02	2.63, 2.74, 3.05, 3.20
***E_A_***	2.16	1.84	1.44	0.93	0.86
***E_B_***	–	2.21	2.29	2.36	2.38
***E_IB_***	–	0.37	0.85	1.43	1.52

**Table 3:** Bader charge analysis for the lowest energy configurations of Pd*_n_* (*n* = 1–5) clusters supported by the MoS_2_ ML.

Configurations	Pd_1_	Pd_2_	Pd_3_	Pd_4_	Pd_5_
first layer	9.74	9.80(2)	9.82(3)	9.80(2), 9.82	9.78(2), 9.79, 9.89
second layer	–	–	–	10.14	10.15
Bader charge	0.26	0.40	0.54	0.44	0.61

**Table 4:** Calculated work function (*W*), *p*-SBH (*Φ_P_*), dipole moment (*D_i_*), and variations of ionization energy (Δ*I*) upon the adsorption of the lowest energy Pd*_n_* (*n* = 1–5) clusters on the MoS_2_ ML.

Configurations	Pd_1_	Pd_2_	Pd_3_	Pd_4_	Pd_5_
*W* (eV)	5.01	4.73	4.62	4.46	4.38
*Φ_P_* (eV)	0.52	0.82	1.00	1.18	1.27
Δ*I* (eV)	0.17	0.16	0.08	0.06	0.05
*D_i_* (eÅ^−1^)	0.35	0.57	0.80	0.64	0.88

## References

[1] Xu M., Liang T., Shi M., Chen H. (2013). Graphene-Like Two-Dimensional Materials. Chem. Rev..

[2] Splendiani A., Sun L., Zhang Y., Li T., Kim J., Chim C.Y., Galli G., Wang F. (2010). Emerging Photoluminescence in Monolayer MoS_2_. Nano Lett..

[3] Chen M., Nam H., Wi S., Priessnitz G., Gunawan I.M., Liang X. (2014). Multibit Data Storage States Formed in Plasma-Treated MoS_2_ Transistors. ACS Nano.

[4] Yu Z., Ong Z.Y., Li S., Xu J.B., Zhang G., Zhang Y.W., Shi Y., Wang X. (2017). Analyzing the Carrier Mobility in Transition-Metal Dichalcogenide MoS_2_ Field-Effect Transistors. Adv. Funct. Mater..

[5] Chen S., Wang H., Lu S., Xiang Y. (2016). Monolayer MoS_2_ film supported metal electrocatalysts: A DFT study. RSC Adv..

[6] Fu N., Hu Y., Shi S., Ren S., Liu W., Su S., Zhao B., Weng L., Wang L. (2018). Au nanoparticles on two-dimensional MoS_2_ nanosheets as the photoanode for efficient photoelectron chemical miRNA detection. Analyst.

[7] Burman D., Santra S., Pramanik P., Guha P.K. (2018). Pt decorated MoS_2_ nanoflakes for ultrasensitive resistive humidity sensor. Nanotechnology.

[8] Li X.D., Fang Y.M., Wu S.Q., Zhu Z.Z. (2015). Adsorption of alkali, alkaline-earth, simple and 3d transition metal, and nonmetal atoms on monolayer MoS_2_. AIP ADV.

[9] Shi Y., Huang J., Jin L., Hsu Y., Yu S.F., Li L.J., Yang H.Y. (2013). Selective Decoration of Au Nanoparticles on Monolayer MoS_2_ Single Crystals. Sci. Rep..

[10] Guo Y., Dun C., Xu J., Li P., Huang W., Mu J., Hou C., Hewitt C.A., Zhang Q., Li Y. (2018). Wearable Thermoelectric Device Based on Au Decorated Two-Dimensional MoS_2_. ACS Appl. Mater. Interfaces.

[11] Wang X., Tian H., Zhao H., Zhang T., Mao W., Qiao Y., Pang Y., Li Y., Yang Y., Ren T. (2017). Interface Engineering with MoS_2_–Pd Nanoparticles Hybrid Structure for a Low Voltage Resistive Switching Memory. Small.

[12] Huang X., Zeng Z., Bao S., Wang M., Qi X., Fan Z., Zhang H. (2013). Solution-phase epitaxial growth of noble metal nanostructures on dispersible single-layer molybdenum disulfide nanosheets. Nat. Commun..

[13] Song B., He K., Yuan Y., Sharifi-Asl S., Cheng M., Lu J., Saidi W.A., Yassar Reza S. (2018). In Situ Study of Nucleation and Growth Dynamics of Au Nanoparticles on MoS_2_ Nanoflakes. Nanoscale.

[14] Saidi W.A. (2015). Density Functional Theory Study of Nucleation and Growth of Pt Nanoparticles on MoS_2_ (001) Surface. Cryst. Growth Des..

[15] Jiang C., Wang Y., Zhang Y., Wang H., Chen Q., Wan J. (2018). Robust Half-Metallic Magnetism in Two-Dimensional Fe/MoS_2_. J. Phys. Chem. C.

[16] Šljivančanin Ž., Belić M. (2017). Graphene/MoS_2_ heterostructures as templates for growing two-dimensional metals: Predictions from ab initio calculations. Phys. Rev. Mater..

[17] Schulman D.S., Arnold A.J., Das S. (2018). Contact engineering for 2D materials and devices. Chem. Soc. Rev..

[18] Fontana M., Deppe T., Boyd A.K., Rinzan M., Liu A.Y., Paranjape M., Barbara P. (2013). Electron-hole transport and photovoltaic effect in gated MoS_2_ Schottky junctions. Sci. Rep..

[19] Dong H., Gong C., Addou R., McDonnell S., Azcatl A., Qin X., Wang W., Wang W., Hinkle C.L., Wallace R.M. (2017). Schottky barrier height of Pd/MoS_2_ contact by large area photoemission spectroscopy. ACS Appl. Mater. Interfaces.

[20] Perdew J.P., Burke K., Ernzerhof M. (1996). Efficient iterative schemes for ab initio total-energy calculations using a plane-wave basis set. Phys. Rev. Lett..

[21] Kresse G., Furthmüller J. (1996). Projector augmented-wave method. Phys. Rev. B Condens. Matter Mater. Phys..

[22] Blochl P.E. (1994). Projector augmented-wave method. Phys. Rev. B Condens. Matter.

[23] Monkhorst H.J., Pack J.D. (1976). Special points for Brillouin-zone integrations. Phys. Rev. B Solid State.

[24] Henkelman G., Uberuaga B.P., Jónsson H. (2000). A climbing image nudged elastic band method for finding saddle points and minimum energy paths. J. Chem. Phys..

[25] Henkelman G., Jónsson H. (2000). Improved tangent estimate in the nudged elastic band method for finding minimum energy paths and saddle points. J. Chem. Phys..

[26] Wu P., Yin N., Li P., Cheng W., Huang M. (2017). The adsorption and diffusion behavior of noble metal adatoms (Pd, Pt, Cu, Ag and Au) on a MoS_2_ monolayer: A first-principles study. Phys. Chem. Chem. Phys..

[27] Li H., Huang M., Cao G. (2017). Magnetic properties of atomic 3d transition-metal chains on S-vacancy-line templates of monolayer MoS_2_: Effects of substrate and strain. J. Mater. Chem. C.

[28] Matte H.S.S.R., Gomathi A., Manna A.K., Late D.J., Datta R., Pati S.K., Rao C.N.R. (2010). MoS_2_ and WS_2_ Analogues of Graphene. Angew. Chem. Int. Ed..

[29] Mak K.F., Lee C., Hone J., Shan J., Heinz T.F. (2010). Atomically thin MoS_2_: A new direct-gap semiconductor. Phys. Rev. Lett..

[30] Saidi W.A. (2015). Trends in the Adsorption and Growth Morphology of Metals on the MoS_2_(001) Surface. Cryst. Growth. Des..

[31] Wang Y., Li Y., Chen Z. (2015). Not your familiar two-dimensional transition metal disulfide: Structural and electronic properties of the PdS_2_ monolayer. J. Mater. Chem. C.

[32] Wu P., Cao G., Tang F., Huang M. (2013). First-principles study of small palladium clusters on NiAl(110) alloy surface. Physica E.

[33] Wang B., Yoon B., König M., Fukamori Y., Esch F., Heiz U., Landman U. (2012). Size-Selected Monodisperse Nanoclusters on Supported Graphene: Bonding, Isomerism, and Mobility. Nano Lett..

[34] Kwon S., Choi S.H., Kim Y.J., Yoon I.T., Yang W. (2018). Proton beam flux dependent work function of mono-layer MoS_2_. Thin Solid Films.

[35] Dong Y.F., Wang S.J., Mi Y.Y., Feng Y.F., Huan A.C.H. (2006). First-principles studies on initial growth of Ni on MgO(001) surface. Surf. Sci..

[36] Topham B.J., Kumar M., Soos Z.G. (2011). Profiles of Work Function Shifts and Collective Charge Transfer in Submonolayer Metal–Organic Films. Funct. Mater..

[37] Monti O.L.A. (2012). Understanding Interfacial Electronic Structure and Charge Transfer: An Electrostatic Perspective. J. Phys. Chem. Lett..

[38] Piacenza M., D’Agostino S., Fabiano E., Sala F.D. (2009). Ab initio depolarization in self-assembled molecular monolayers: Beyond conventional density-functional theory. Phys. Rev. B.

[39] Xu L., Henkelman G., Campbell C.T., Jónsson H. (2005). Small Pd Clusters, up to the Tetramer At Least, Are Highly Mobile on the MgO(100) Surface. Phys. Rev. Lett..

[40] Gong C., Huang C., Miller J., Cheng L., Hao Y., Cobden D., Kim J., Ruoff R.S., Wallace R.M., Cho K. (2013). Metal Contacts on Physical Vapor Deposited Monolayer MoS_2_. ACS Nano.

